# Antimicrobial and Antioxidant Activities of New Metal Complexes Derived from 3-Aminocoumarin

**DOI:** 10.3390/molecules16086969

**Published:** 2011-08-15

**Authors:** Abdul Amir H. Kadhum, Abu Bakar Mohamad, Ahmed A. Al-Amiery, Mohd S. Takriff

**Affiliations:** 1 Department of Chemical and Process Engineering, Faculty of Engineering and Built Environment, Universiti Kebangsaan Malaysia, Bangi, Selangor 43600, Malaysia; 2 Biotechnology Division, Applied Science Department, University of Technology, Baghdad 10066, Iraq

**Keywords:** 3-aminocoumarin, antimicrobial, complexes, DPPH, scavenging

## Abstract

3-Aminocoumarin (**L**) has been synthesized and used as a ligand for the formation of Cr(III), Ni(II), and Cu(II) complexes. The chemical structures were characterized using different spectroscopic methods. The elemental analyses revealed that the complexes where M=Ni(II) and Cu(II) have the general formulae [ML_2_Cl_2_], while the Cr(III) complex has the formula [CrL_2_Cl_2_]Cl. The molar conductance data reveal that all the metal chelates, except the Cr(III) one, are non-electrolytes. From the magnetic and UV-Visible spectra, it is found that these complexes have octahedral structures. The stability for the prepared complexes was studied theoretically using Density Function Theory. The total energy for the complexes was calculated and it was shown that the copper complex is the most stable one. Complexes were tested against selected types of microbial organisms and showed significant activities. The free radical scavenging activity of metal complexes have been determined by measuring their interaction with the stable free radical DPPH and all the compounds have shown encouraging antioxidant activities.

## 1. Introduction

Drug resistance has become a growing problem in the treatment of infectious diseases caused by bacteria and fungi [1]. The serious medical problem of bacterial and fungal resistance and the rapid rate at which it develops has led to increasing levels of resistance to classical antibiotics [2,3], and the discovery and development of effective antibacterial and antifungal drugs with novel mechanisms of action have thus become urgent tasks for infectious disease research programs [4]. Coumarins present a variety of bioactivities, including anticoagulant, estrogenic, dermal photosensitizing, antimicrobial, vasodilator, molluscicidal, antihelmintic, sedative and hypnotic, analgesic and hypothermic actions [5,6,7,8,9,10,11,12,13,14,15,16]. In addition, coumarins have been shown to inhibit *N*-methyl-*N*-nitrosourea, aflatoxin B1 and 7,12-dimethylbenz(a)anthracene-induced mammary carcinogenesis in rats [17,18]. More recently, coumarin derivatives had been evaluated in the treatment of human immunodeficiency virus, due to their ability to inhibit human immunodeficiency virus [19,20]. Since the late 1980s, a number of *in vitro* and *in vivo* studies have investigated the possible use of coumarins in the treatment of cancer [21]. Coumarin derivatives exhibit not only excellent biological and medical activities [22], but also have the superior thermal stability and outstanding optical properties, including extended spectral responses, high quantum yields, and superior photostability. Optical applications of these compounds, such as laser dyes, nonlinear optical chromophore, fluorescent whiteners, fluorescent probes, polymer science, optical recording and solar energy collectors, have been widely investigated [23,24,25,26,27,28]. More importantly, coumarin dyes are used as blue, green and red dopants in organic light-emitting diodes (OLEDs) [29,30]. Based on the structure of coumarin where there exists delocalization of π-electrons (resonance effect), the potential for this molecule to be used as chemical inhibitor can be established [31].

3-Aminocoumarin derivatives have been found to also possess a wide range of biological activities, including CNS depressant, [32] antibacterial, [33] antiallergic [34] and insect-growth regulatory effects [35]. Medicinal metal complexes have become an interesting research area since the discovery of cisplatin [36]. Since then, many complexes have been synthesized and tested on a number of biological systems. Copper complexes are known to have a broad spectrum of biological action [37]. 

The preparation of 3-aminocoumarin and its use as a ligand for the formation of Cr(III), Ni(II), and Cu(II) complexes, is presented in this study. The chemical structures of the newly synthesized complexes were confirmed. The microbial activities of all synthesized compounds and their *in vitro* antioxidant activities were also investigated.

## 2. Results and Discussion

### 2.1. Chemistry

**Figure 1:**
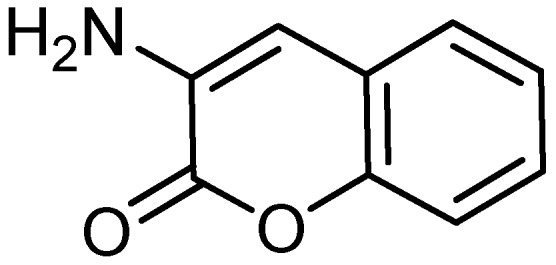
The structure of 3-aminocoumarin.

3-Aminocoumarin (Figure 1) is the key intermediate for the metal complexes synthesized in this work.

**Figure 2:**
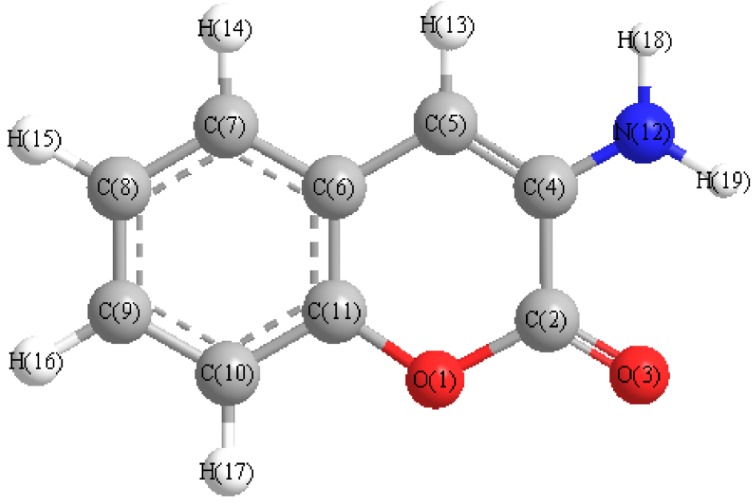
The 3d structure of 3-aminocoumarin.

The data for the minimized geometry and the 3d-geometrical structure of 3-aminocoumarin (Figure 2) show that the atomic charges have been affected by the presence of the ring substituent, as shown in Table 1.

**Table 1:** The atomic charges of 3-aminocoumarin.

Atom	Charge	Atom	Charge	Atom	Charge
**O(1)**	−0.0576918	C(7)	−0.115268	H(13)	0.0181674
**C(2)**	0.546493	C(8)	−0.0752691	H(14)	0.0223331
**O(3)**	−0.580016	C(9)	−0.101239	H(15)	0.0253347
**C(4)**	0.144636	C(10)	−0.110194	H(16)	0.0249427
**C(5)**	−0.359074	C(11)	0.197216	H(17)	0.0281505
**C(6)**	0.00167107	N(12)	0.205349	H(18)	0.092316
**H(19)**	0.0921441	−	−	−	

The data obtained show that the highest atomic charge in the 3-aminocoumarin molecule is located at [O(3)-0.580016], while the next highest charge value are at [C(5)-0.359074] and [C(7)-0.115268]. These data show clearly that oxygen atom [O(3)] is the most reactive toward the bonding with the metal. The determined bond angle, twist angle, 3D geometrical structure (Figure 2) and stereochemistry [C(4)-C(5):(E)], indicate that this molecule is planar.

**Scheme 1:**
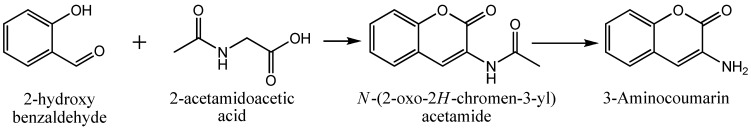
Formation of the ligand.

The synthesis of 3-aminocoumarin was performed by vigorously refluxing 2-hydroxybenzaldehyde with 2-acetamidoacetic acid, using piperidine as the strong base. The end of the reaction involves the removal of the acetyl group and the production of 3-aminocoumarin in 17% yield (Scheme 1).

**Scheme 2:**
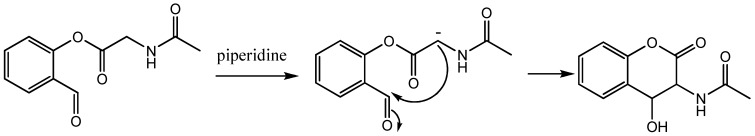
Reaction mechanism of formation of the ligand.

The mechanism of the reaction may be explained by a carbanion mechanism (Scheme 2).

The complexes were synthesized by the reactions of 3-aminocoumarin with the metal ions, in which the ligand behaves as a bidentate ligand through its oxygen and nitrogen atoms. The analytical data of these complexes are presented in Table 2. 

**Table 2:** Analytical data for the metal complexes.

No.	Compounds	M:L	M.P. °C	Yield %	Elemental analysis (calculated)				Elemental analysis (found)			
					C%	H%	N%	M%	C%	H%	N%	M%
**C_1_**	[Cr(L)_2_Cl_2_]Cl	1:2	189	40	44.98	2.94	5.83	10.82	45.02	2.42	5.30	10.65
**C_2_**	[Ni(L)_2_Cl_2_]	1:2	221d	55	47.84	3.12	6.20	12.99	47.59	3.07	6.09	12.53
**C_3_**	[Cu(L)_2_Cl_2_]	1:2	209d	67	47.33	3.09	6.13	13.91	46.98	2.80	5.92	13.99

All the complexes are fairly stable and can be stored for long periods at room temperature. The solid colored complexes are stable towards heat, air and moisture, and they are generally soluble in common organic solvents such as dimethylformamide or dimethylsulfoxide. The stability for the prepared complexes was studied theoretically using Density Function Theory (DFT). The total energy for the complexes was calculated and it was shown that the copper complex is the most stable and the chromium complex is the least stable as follows: Cu(II) > Ni(II) > Cr(III). In all our work on metal complexes (in which we have used different ligands with the ion metals Cu, Ni, and Cr), we found out that the copper complex is consistently the most stable.

**Figure 3:**
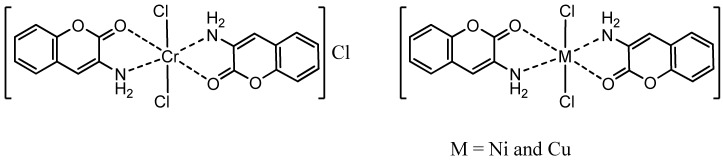
The proposed structure for the complexes.

#### 2.1.1. Elemental Analysis

The compositions of the complexes are summarized in Table 2. The analytical data of the complexes are consistent with the proposed molecular structures (Figure 3), assuming molar metal to ligand ratios of 1:2. The C, H, N and M contents (both theoretically calculated values and actual values) are in accordance with the formula ML_2_Cl_2_ indicating that the 3-aminocoumarin complexes are neutral, except for the Cr(III) complex with formula [ML_2_Cl_2_]Cl (Table 2). This can be explained by the absence of any deprotonating agent during the synthesis. 

#### 2.1.2. Infrared Spectra

A study and comparison of the infrared spectra of 3-aminocoumarin and its complexes imply that 3-aminocoumarin is bidentate, with the carbonyl oxygen and amine nitrogen as the two coordination sites. In the infrared spectra of the complexes a considerable negative shift of the C=O group is observed, indicating a decrease in the stretching force-constant of the C=O bond as a consequence of coordination through the carbonyl oxygen atom. The carbonyl band for the parent 3-aminocoumarin ligand was located at 1709 cm^−1^, but for the Cr(III), Ni(II) and Cu(II) complexes these peaks were shifted to lower frequencies, namely 1691 cm^−1^, 1701 cm^−1^, and 1685 cm^−1^, respectively. The NH_2 _bands of 3-aminocoumarin were at 3407 and 3299 cm^−1^, but for the complexes the NH_2_ bands also shift to lower frequencies [Cr-complex ones were at 3315 and 3201 cm^−1^, Ni-complex at 3310 and 3181 cm^−1^ and finally the Cu-complex ones were at 3326 and 3217 cm^−1^, respectively] [38]. Additional evidence for the coupling between the ligand and metals ion were the M-N and M-O bands occurring at 455-467 cm^-1^and 486-503 cm^−1^, respectively (Table 3).

**Table 3:** FT-IR (cm^−1^) bands of metal complexes.

NO.	N–H	C=O	M–Cl	νM–N	νM–O
**C_1_**	3315, 3201	1691	343	467	503
**C_2_**	3310, 3181	1701	330	455	501
**C_3_**	3326, 3217	1685	330	459	486

#### 2.1.3. ^1^H-NMR Spectra

The ^1^H-NMR spectrum of the ligand (L) showed characteristic signals due to the N-H protons at 7.97 ppm. Moreover the peaks observed between 7.40 ppm and 7.61 ppm were assigned to the C–H_aromatic_ protons of the ligand. The signal at 6.11 ppm may be assigned to the =C–H_vinyl_ proton.

#### 2.1.4. Molar Conductance

The molar conductance values of all the complexes determined in nitrobenzene at room temperature are given in Table 4. The value for the Cr(III) complex indicates that one chloride ion is present outside the coordination sphere. The molar conductance values Ni(II) and Cu(II) complexes are quite low to correspond to an ionic complex; hence, these complexes are considered to be neutral and the chloride ions are assumed to be situated within the coordination sphere.

**Table 4:** Physical data of the synthesized compounds.

No.	λ_max_ cm^−1^	Magnetic moment µ (B.M.)	ʌ ohm ^1^ cm^2^ mol^−1^	Structure
**C_1_**	15217, 20112, 32125	4.1	90	Octahedral
**C_2_**	15890, 31915	2.6	20	Octahedral
**C_3_**	12117, 15812, 22076	1.6	22	Octahedral

#### 2.1.5. Magnetic Moment and UV-Vis Spectra

The ultraviolet spectrum of the synthesized 3-aminocoumarin showed two absorption bands. The position of the first band, which represents the (π → π*) transition, was at 239 nm, while the second band (which has higher intensity than the first one due to the conjugated system) appeared at 289 nm and represents the (n → π*) transition. Generally, the bands of the newly synthesized complexes are either shifted to shorter or longer wavelengths than those of 3-aminocoumarin, but the high intensity of the band is an indication for complex formation. 

In these complexes the bands observed over 300 nm could be assigned to nitrogen-metal charge transfer absorption. The electronic absorption bands for the ligand and complexes are classified into two distinct groups, first those that belong to ligand transitions appeared in the UV region, while d-d transitions appeared in the visible region. These transitions are releted to the structures of the complexes (Table 4). The Cr(III) complex showed three bands with absorbance maxima at 15,217 cm^−1^, 20,112 cm^−1^ and 32,125 cm^−1^ which were assigned to the ^4^A_2_g(F) → ^4^T_2_g_(F)_, ^4^A_2_g_(F)_ → ^4^T_1_g_(F)_, ^4^A_2_g_(F)_ → ^4^T_1_g_(P)_ absorption bands, respectively. These transitions, combined with the measured 4.1 B.M. magnetic moment, suggest a high-spin octahedral geometry for the Cr(III) complex.

The nickel complex is paramagnetic, with a room temperature magnetic moment of 2.6 B.M., which is consistent with an octahedral field. The electronic absorption spectrum showed two absorption bands at 15,890 cm^−1^ and 31,915 cm^−1^ which are considered to correspond to ^3^A_2_g_(F)_ → ^3^T_1_g and ^3^A_2_g_(F)_ → ^3^T_1_g_(P)_ (Table 4), respectively. 

The room temperature magnetic moment of 1.6 B.M. indicates an octahedral structure for the Cu(II) ion complex. The electronic absorption spectrum of Cu(II) complex shows three bands at 12,117 cm^−1^, 15,812 cm^−1^ and 22,076 cm^−1^, assigned to ^2^B_1_g → ^2^A_2_g, ^2^B_1_g → ^2^B_2_g and ^2^B_1_g → ^2^Eg, respectively [38].

#### 2.1.6. Suggested Stereostructures of the Complexes

The proposed structures of complexes based on the above mentioned data (UV-Vis, IR, and NMR spectra, conductivity, molar ratio and magnetic properties) are depicted in Figure 3. The chlorides in the metal complexes of Ni(II) and Cu(II), are in the octahedral coordination sphere, but for the Cr(III) complex, the chlorides are in spherical coordination and the third chloride is in the outer of the spherical coordination, as implied by the molar conductance.

### 2.2. Pharmacology

#### 2.2.1. Antibacterial Activity

The antibacterial screening data show that the complexes exhibit antimicrobial properties, and we note that the metal chelates exhibit more inhibitory effects than the parent 3-aminocoumarin ligand. The increased activity of the metal chelates can be explained on the basis of chelation theory [39]. It is known that chelation tends to make the ligand act as powerful and potent bactericidal agents, thus killing more of the bacteria than the ligand alone. It is observed that, in a complex, the positive charge of the metal is partially shared with the donor atoms present in the ligands, and there may be *π*-electron delocalization over the whole chelating space [40]. This increases the lipophilic character of the metal chelate and favours its permeation through the lipoid layer of the bacterial membranes. The increased lipophilic character of these complexes seems to be responsible for their enhanced potent antibacterial activity. It may be suggested that these complexes deactivate various cellular enzymes, which play a vital role in various metabolic pathways of these microorganisms. It has also been proposed that the ultimate action of the toxicant is the denaturation of one or more proteins of the cell, which as a result, impairs normal cellular processes. There are other factors which also increase the activity, which are solubility, conductivity, and bond length between the metal and the ligand.

**Figure 4:**
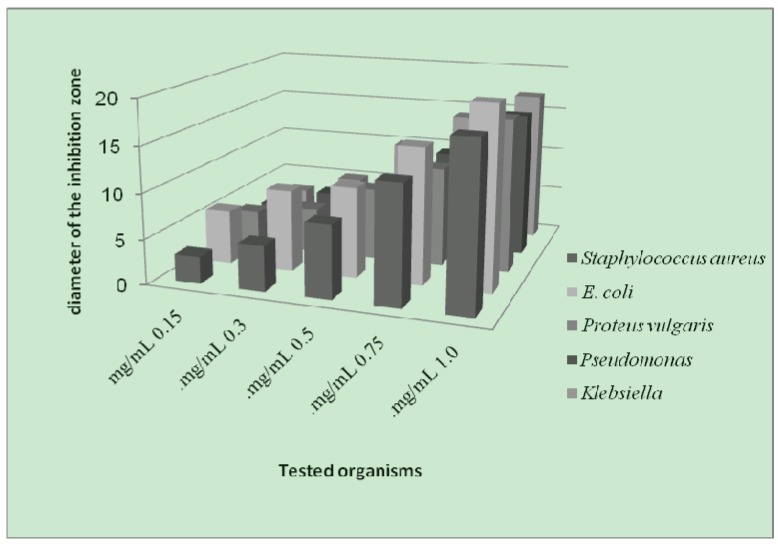
Effect of the Cr-complex toward test organisms.

**Figure 5:**
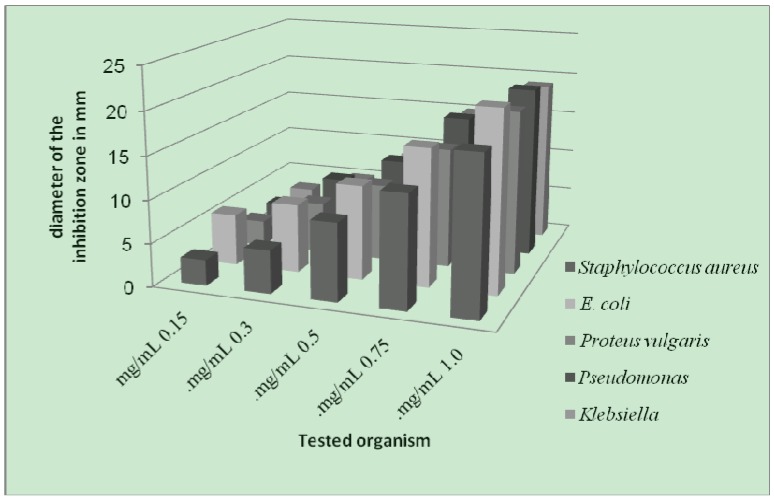
Effect of Ni-complex toward test organisms.

**Figure 6:**
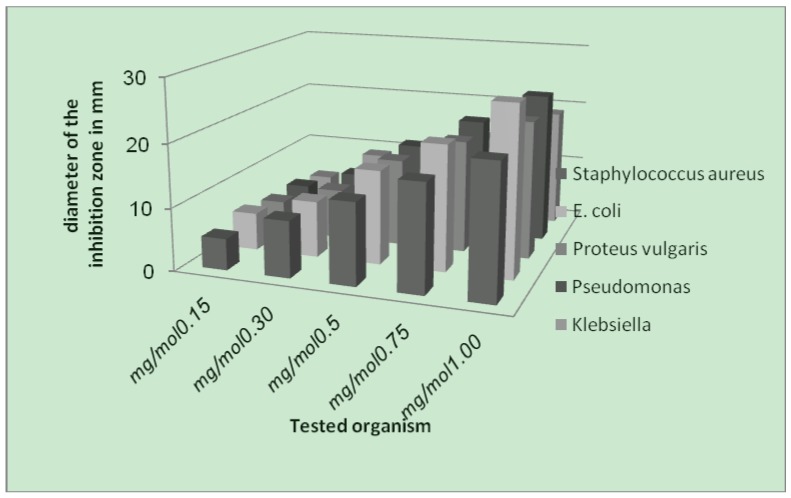
Effect of Cu-complex toward test organisms.

As a result from the study of antibacterial of the prepared metal complexes (Figure 4, Figure 5 and Figure 6), the following conclusions may be stated:

(1). Generally, the result of prepared complexes exhibited antibacterial activity toward *E. coli* bacteria was more than the inhibition on other types of bacteria.
(2). The copper complex has more activity toward all the kinds of tested bacteria, compared to other complexes. The stability of the copper complex and the coupling with the ligand may be the reasons for this activity against bacteria.

#### 2.2.2. Antifungal Activities

**Figure 7:**
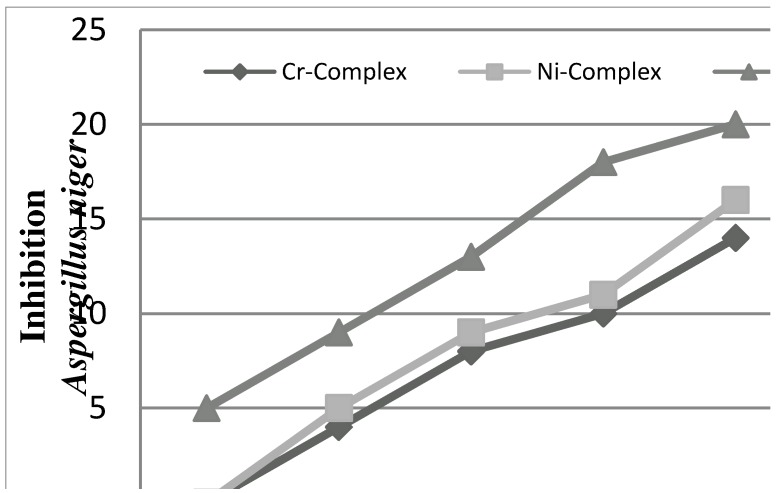
The effect of complexes toward *Aspergillus niger*.

**Figure 8:**
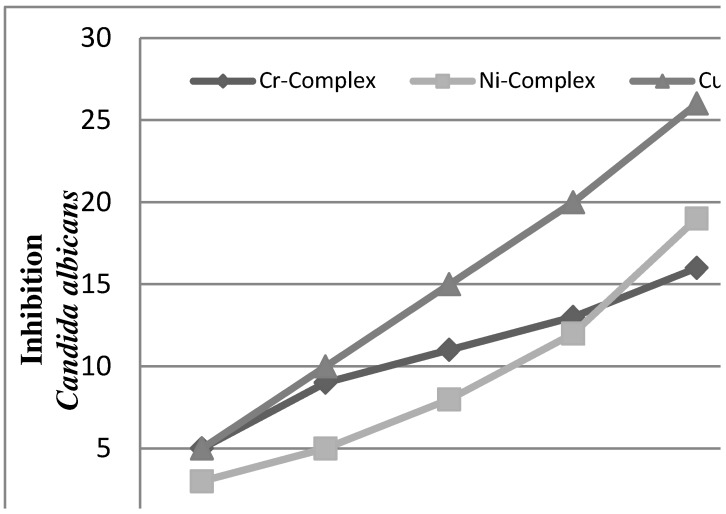
The effect of complexes toward *Candida albicans*.

*In vitro* antifungal screening effects of the investigated compounds were tested against some fungal species (*Aspergillus niger and Candida albicans*). The Cu(II) complex was found to exhibit antifungal activity against all the fungi in this study (Figure 7 and Figure 8).

#### 2.2.3. Radical Scavenging Activity

The 2,2′′-diphenyl-1-picrylhydrazyl (DPPH) radical assay provides an easy and rapid way to evaluate the antiradical activities of antioxidants. Determination of the reaction kinetic types DPPHH is a product of the reaction between DPPH• and an antioxidant:

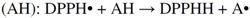



The reversibility of the reaction is evaluated by adding DPPHH at the end of the reaction. If there is an increase in the percentage of remaining DPPH• at the plateau, the reaction is reversible, otherwise it is a complete reaction.

**Figure 9:**
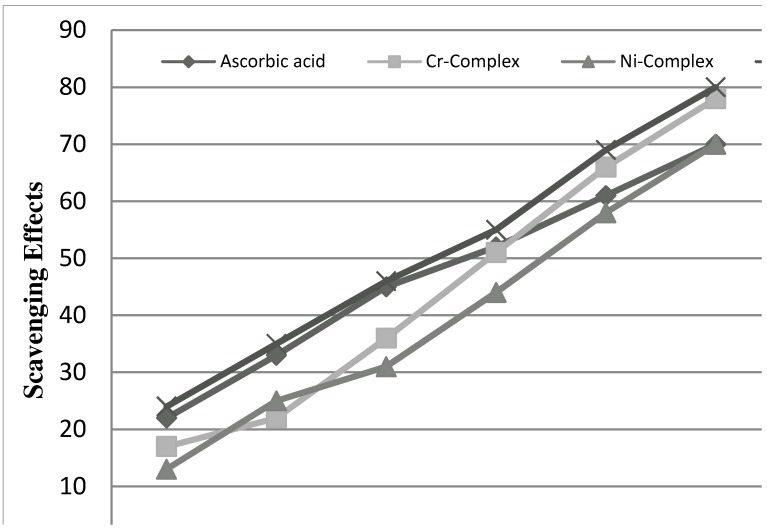
Scavenging effect of metal complexes, and ascorbic acid at different concentrations (15, 30, 45, 60, 80 and 100 μg/mL).

DPPH was used as stable free radical electron accepts or hydrogen radical to become a stable diamagnetic molecule [41]. DPPH is a stable free radical containing an odd electron in its structure and usually used for detection of the radical scavenging activity in chemical analysis. [42]. The reduction capability of DPPH radicals was determined by decrease in its absorbance at 517 nm induced by antioxidants. [43]. The graph was plotted with percentage scavenging effects on the y-axis and concentration (µg/mL.) on the x-axis. The scavenging ability of the metal complexes was compared with ascorbic acid as a standard. The metal complexes showed good activities as a radical scavenger compared with ascorbic acid, Figure 9. These results were in agreement with previous metallic complexes studies where the ligand has the antioxidant activity and it is expected that the metal moiety will increase its activity [44,45,46].

## 3. Experimental

### 3.1. General

All chemicals used in this work were of reagent grade (supplied by either Sigma-Aldrich or Fluka) and used without farther purifications. The FTIR spectra were recorded in the (4000–200) cm^−1^ range on cesium iodide disks using a Shimadzu FTIR 8300 Spectrophotometer. Proton NMR spectra were recorded on Bruker-DPX 300 MHz spectrometer with TMS as internal standard. The UV-Visible spectra were measured in ethanol using a Shimadzu UV-Vis. 160A spectrophotometer in the range (200–1000) nm. Magnetic susceptibility measurement for complexes was obtained at room temperature using a Magnetic Susceptibility Balance Model MSB-MKI. The flame atomic absorption of a Shimadzu AA-670 elemental analyzer was used for metal determination. Elemental micro analysis was carried out using a CHN elemental analyzer model 5500-Carlo Erba instrument. A Gallenkamp M.F.B.600.010 F melting point apparatus was used to measure the melting points of all the prepared compounds.

### 3.2. Chemistry

#### 3.2.1. Synthesis of the Ligand

A mixture of 2-acetamidoacetic acid (58.5 g, 0.5 mol) and 2-hydroxybenzaldehyde (92.7 g, 0.76 mol) in acetic anhydride (0.5 mL, 0.053 mol) with few drops of piperidine was refluxed at 130 °C for 8 hours. After cooling to room temperature the product is separated out and washed with diethyl ether several times, dried and then recrystallized from ethanol to give *N*-(2-oxo-2*H*-chromen-3-yl)acetamide in 85% yield. The latter (5 g, 0.02 mol) was refluxed in ethanol (25 mL), containing conc. hydrochloric acid (2 mL) for 4 hours. The crystallized compound (impure 3-aminocoumarin) mixture was cooled, poured on to ice before neutralization with sodium bicarbonate. The solid was separated out, filtered and left to dry. 3-Aminocoumarin was recrystallized from ethanol, giving a pale yellow powder, yield 17%, m.p. 129 °C (lit. [47] 130 ^o^C); ^1^H-NMR (CDCl_3_): *δ* 7.97 (s, NH_2_), *δ* 6.11 (s, 1H) for –C=C-H), *δ* 7.40-7.61 (m, 1H) for aromatic ring); IR: 3407 and 3299 cm^−1^ (NH_2_, amine), 1709 cm^−1^ (C=O, lactone); Anal. Calcd. for C_9_H_7_NO_2_: C 67.07%, H 4.38%, N 8.69%. Found: C 66.98%, H 4.27%, N 8.58%.

#### 3.2.2. Synthesis of the Complexes

Metal salts (CrCl_3_·6H_2_O, NiCl_2_·6H_2_O and CuCl_2_·2H_2_O; 5 mmol) in hot ethanol (20 mL) were mixed with hot ethanolic solution of the 3-aminocoumarin (1.61 g, 10 mmol) and refluxed for 4 hours. On cooling the contents, the colored complexes were separated out. The products were filtered [48], washed with cold 50% ethanol and dried in vacuum over P_4_O_10_. Purity of the complexes was checked by Thin Layer Chromatography (TLC). The TLC plates (20 × 10 cm) were recoated silica gel on aluminum 60_F_ – 254, with a stationary phase thickness of about 0.5 mm. Five μL each of test solution was applied on each plate. The TLC plate was placed in a saturated chromatographic tank containing an ethyl acetate-methanol-acetone (25:25:50) solvent system, R*_f_* values for Cu(II), Ni(II) and Cr(III) were 0.31, 3.0 and 4.3, respectively.

#### 3.2.3. Study of Complex Formation in Solution

The complexes of the 3-aminocoumarin with metal ions were studied in dimethylformamide (DMF), in order to determine the M:L ratio in the complex following the molar ratio method. Several series of solutions were prepared having constant concentration (10^−3^ M) of the metal ion and (L). The M:L ratios were determined from the relationship between the absorption of light [49] and the M:L mole ratio. The results of complexes formation in solution were listed in Table 2.

### 3.3. Pharmacology

#### 3.3.1. Evaluation of Antibacterial Activities

The *in vitro* antibacterial effects of the complexes were evaluated against a sp. of Gram-positive bacteria (*Staphylococcus aureus*) and four Gram-negative bacteria (*Escherichia coli, Pseudomonas aeruginosa*, *Klebsiella pneumonia and Proteus vulgaris*) by the disc diffusion method [50] using nutrient agar medium. The bacteria were sub-cultured in the agar medium and were incubated for 24 h. at 37 °C. The discs having a diameter of 5 mm, then soaked in the test solutions (Sterile filter paper discs, Whatman No. 1.0) with the appropriate equivalent amount of the metal complexes dissolved in sterile dimethyl sulphoxide (DMSO) at concentrations of 1–10 mg/disc) and were placed in Petri dishes on an appropriate medium previously seeded with organisms and stored in an incubator for the above mentioned period of time. The inhibition zone around each disc was measured and the results recorded in the form of inhibition zones (diameter, mm). To clarify any effect of DMSO on the biological screening, separate studies were carried out using DMSO as control and it showed no activity against any bacterial strains. 

#### 3.3.2. Evaluation of Antifungal Assay

Antifungal activity [51,52], based on the determined growth inhibition rates of the mycelia of strain (*Aspergillus niger* and *Candida albicans*) in Potato Dextrose Broth medium (PDB). Under aseptic conditions, one mL of spore suspension (5 × 10^6^ cfu/mL) of tested fungi was added to 50 mL PDB medium in a 100 mL Erlenmeyer flask. Appropriate volumes of tested metal complexes were added to produce concentrations ranging from 10 to 100 μg mL^−1^. Flasks were incubated at 27 ± 1 °C in the dark for 5 days and then the mycelium was collected on filter papers. The filter papers were dried to constant weight and the level of inhibition, relative to the control flasks was calculated from the following formula:

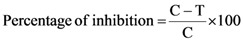

where T = weight of mycelium from test flasks and C = weight of mycelium from control flasks.

#### 3.3.3. Evaluation of Antioxidant Activity

Stock solution (1 mg/mL) was diluted to final concentrations of 20–100 μg/mL. Ethanolic DPPH solution (1 mL, 0.3 mmol) was added to sample solutions in DMSO (3 mL) at different concentrations (50–300 μg/mL) [53]. The mixture was shaken vigorously and allowed to stand at room temperature for 30 min. The absorbance was then measured at 517 nm in a UV-Vis Spectrophotometer. The lower absorbance of the reaction mixture indicates higher free radical scavenging activity. Ethanol was used as the solvent and ascorbic acid as the standard. The DPPH radical scavenger was calculated using the following equation:

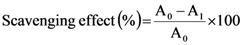

where A_o_ is the absorbance of the control reaction and A_1_ is the absorbance in the presence of the samples or standards. A_0_-A_1_

## 4. Conclusions

In this study, Cr(III), Ni(II) and Cu(II) complexes of 3-aminocoumarin have been successfully synthesized and characterized by using various spectroscopic methods, elemental analysis, magnetic moment and molar conductance studies. The synthesized complexes were tested for antioxidant and antimicrobial activities. Out of these complexes, Cu(II) indicated significant antimicrobial activities as compared to either Cr(III) or Ni(II). In addition the Cu(II) complex is also found to be a superior antioxidant complex as compared to ascorbic acid.
